# ^137^Cs, ^40^K, and K in raw and stir-fried mushrooms from the *Boletaceae* family from the Midu region in Yunnan, Southwest China

**DOI:** 10.1007/s11356-020-09393-w

**Published:** 2020-06-07

**Authors:** Jerzy Falandysz, Ji Zhang, Michał Saniewski

**Affiliations:** 1 Environmental Chemistry and Ecotoxicology, University of Gdańsk, 63 Wita Stwosza Street, 80-308 Gdańsk, Poland; 2 Environmental and Computational Chemistry Group, School of Pharmaceutical Sciences, Zaragocilla Campus, University of Cartagena, Cartagena, Colombia; 3 Medicinal Plants Research Institute, Yunnan Academy of Agricultural Sciences, Kunming, 650200 China; 4 Institute of Meteorology and Water Management - Maritime Branch, National Research Institute, 42 Waszyngtona Av., 81-342 Gdynia, Poland

**Keywords:** Boletus, Cooking, Foods, Fungi, Radionuclides

## Abstract

The parallel batches of the same species and geographical origin mushrooms both raw and stir-fried were investigated to get an insight into the content and intake of ^137^Cs, ^40^K, and K from mushroom meals. The *Boletaceae* family species (*Baorangia bicolor*, *Boletus bainiugan*, *Butyriboletus roseoflavus*, *Retiboletus griseus*, *Rugiboletus extremiorientalis*, and *Sutorius magnificus*) were collected from the Midu County (Dali Bai Autonomous Prefecture) in 2018. The activity concentrations of ^137^Cs in the caps of dried raw mushrooms were in the range 14 ± 1 Bq kg^−1^ dry biomass (db) (*R. griseus*) to 34 ± 2 Bq kg^−1^ db (*R. extremiorientalis*), and in stems from 16 ± 1 Bq kg^−1^ db (*B. bicolor* and *B. bainiugan*) to 23 ± 1 Bq kg^-1^ db (*R. extremiorientalis*). The mean activity concentration in the whole fruiting bodies in all six species was 18 ± 4 Bq kg^-1^ db. The activity concentrations of ^137^Cs were roughly the same in both dehydrated materials, stir-fried, and raw mushrooms, while the contents of ^40^K and stable K were around 2- to 3-fold smaller in stir-fried than raw product. The raw and stir-fried mushrooms on a whole (wet) weight basis showed activity concentrations of ^137^Cs in the range from 1.2 to 3.2 Bq kg^−1^ ww (mean 1.9 ± 0.6 Bq kg^−1^ ww) and 6.0 to 9.4 Bq kg^−1^ ww (mean 7.0 ± 1.2 Bq kg^−1^ ww), respectively. Evidently, when expressed on a whole (wet) weight basis, the cooked mushrooms showed on average around 3.5-fold greater activity concentration of ^137^Cs when compared with raw mushrooms. The ^137^Cs, ^40^K, and total K enrichment in stir-frying (in a whole (wet) weight basis for the meal), confronted with the results for dehydrated raw and fried mushrooms, show the direct correlation with loss of mass (largely moisture) during the cooking procedure but not much of ^137^Cs and ^40^K. Edible wild mushrooms from Yunnan were little contaminated with radiocaesium. As assessed, the mean radioactivity dose from natural ^40^K in around 9.3-fold exceeded the dose obtained for artificial ^137^Cs from stir-fried mushroom meals, which both were very low doses.

## Introduction

Mushrooms that grow in the wild are known to be sensitive to contamination with radiocaesium (^134^Cs/^137^Cs) that originates from atmospheric fallout. Historically, the accident at the Chernobyl nuclear power plant in 1986 caused high and long-lasting pollution over significant parts of continental Europe. This included contamination of forest soils with ^137^Cs in many regions, and consequently also of the wild mushrooms that grow in these forests (Betti et al. 2017; Chiaravalle et al. 2018; Falandysz et al. 2015; Grodzynska 2018; Orita et al. 2017; Travnikova et al. 2002; Tucaković et al. 2018). More recently, a failure of the Fukushima Dai-ichi nuclear power plant in also caused pollution with ^137^Cs of mushrooms in a region of Japan (Prand-Stritzko and Steinhauser 2018; Steinhauser et al. 2014). There was negligible impact of this incident on continental Asia, e.g., in the region of southwestern China (Falandysz et al. 2016, 2017, 2018). Soil in Yunnan is considered little polluted with ^137^Cs, and forest topsoil (0–5 cm layer) sampled from the Changning localization (ca. 200 km west of Midu County) in Yunnan in 2016 showed the ^137^Cs activity concentration at level 4.9 ± 0.6 Bq kg^−1^ dry weight (Falandysz et al. 2018).

The mycelial network of fungi rapidly absorbs radiocesium that is available in litter and soil and is able to translocate it to the emerging fruiting bodies soon after deposition of radioactive fallout (Stijve and Poretti 1990). The bulk of ^137^Cs deposited in forest soil is a result of the low radio decay rate (half-life time is 30.17 years) and slow migration down to soil depths that are relevant for incorporation in fungal biological cycles (Tanaka et al. 2018), but the resulting contamination persists for decades. A fraction of the freshly deposited radiocaesium that is not absorbed by the uppermost layers on the forest floor can infiltrate at a faster rate down to lower soil layers (horizon) via pores in the soil structure (Fuji et al. 2014). Thus, it can also be available relatively rapidly for species whose mycelia extend to greater depths in the soil, e.g., the mycelium in some species can extend to depths of 0.5 m (Ingrao et al. 1992).

The soil layer where a fungus has its highest density of mycelium and the extent of depth and space to which the hyphae penetrate within layers depends largely on the species. For ectomycorrhizal fungi, the soil distribution of taxa is likely species-dependent, and if the soil profile is favorable, the hyphae follow the roots of their symbiotic partners to lower depths. For example, the ectomycorrhizal *Amanita strobiliformis* had the highest density in the 6–12 cm layer, and for the saprobic *Agaricus bernardii*, hyphae live at least down to a depth of 30 cm (Borovička et al. 2014). The long-term retention of ^137^Cs in forest topsoil results in prolonged contamination of mushrooms with this nuclide (Falandysz and Borovička 2013).

Susceptibility to nuclide uptake/bio-concentration typically occurs over a wider range of fungal species (Cocchi et al. 2017; Falandysz et al. 2019d; Mietelski et al. 2010; Zalewska et al. 2016) and fruiting bodies both of the same or several species collected in the same season from the same forested area, usually shows a range of different ^137^Cs activity concentrations. The variability in uptake/bio-concentration efficiency of ^134^Cs/^137^Cs between species and its activity concentration in fungal fruiting bodies is also a function of the stable caesium (^133^Cs) status of the mushroom (Yoshida et al. 2000), but this important aspect has been little studied so far. Additionally, within species, the developmental stage (size) of the fruiting body (e.g., for *Amanita muscaria*) can also influence the activity concentration (Falandysz et al. 2019b).

Many regions of the world are habitats to mushrooms that can be foraged in the wild including the Yunnan province of China, which has high biodiversity with nearly 900 edible species (Wu et al. 2010). Twenty years before the nuclear accident at Chernobyl, mushrooms were already recognized as a possible source of ^137^Cs contamination (coming from the nuclear weapon explosions) for humans (Kiefer and Maushart 1965). However it has also been observed that intake through this pathway could be reduced by cooking processes, e.g., blanching or boiling with excess of water, can decrease the mushroom content of artificial ^137^Cs and the alkali metals, e.g., Li and Cs, which leak into the discarded water phase (Consiglio et al. 1990; Daillant et al. 2013; Pankavec et al. 2019; Skibniewska and Smoczyński 1999; Stijve 1994).

In a number of Asian cuisines, stir-frying of foods including mushrooms, in a wok type pan, is a popular method of cooking. Information on the possible effect of stir-frying mushrooms in a wok, on the ^137^Cs, ^40^Potassium (^40^K), and total Potassium (K) content and their intake from such stir-fried mushrooms meals, are not available in the literature. We investigated the potential effect of stir-frying in a moderate volume of oil using traditional Chinese cooking practice, on the content of ^137^Cs, ^40^Potassium (^40^K), and total Potassium (K) in dishes made of six species of mushrooms widely foraged in Yunnan.

## Materials and methods

### Fungal materials

**Fig. 1:**
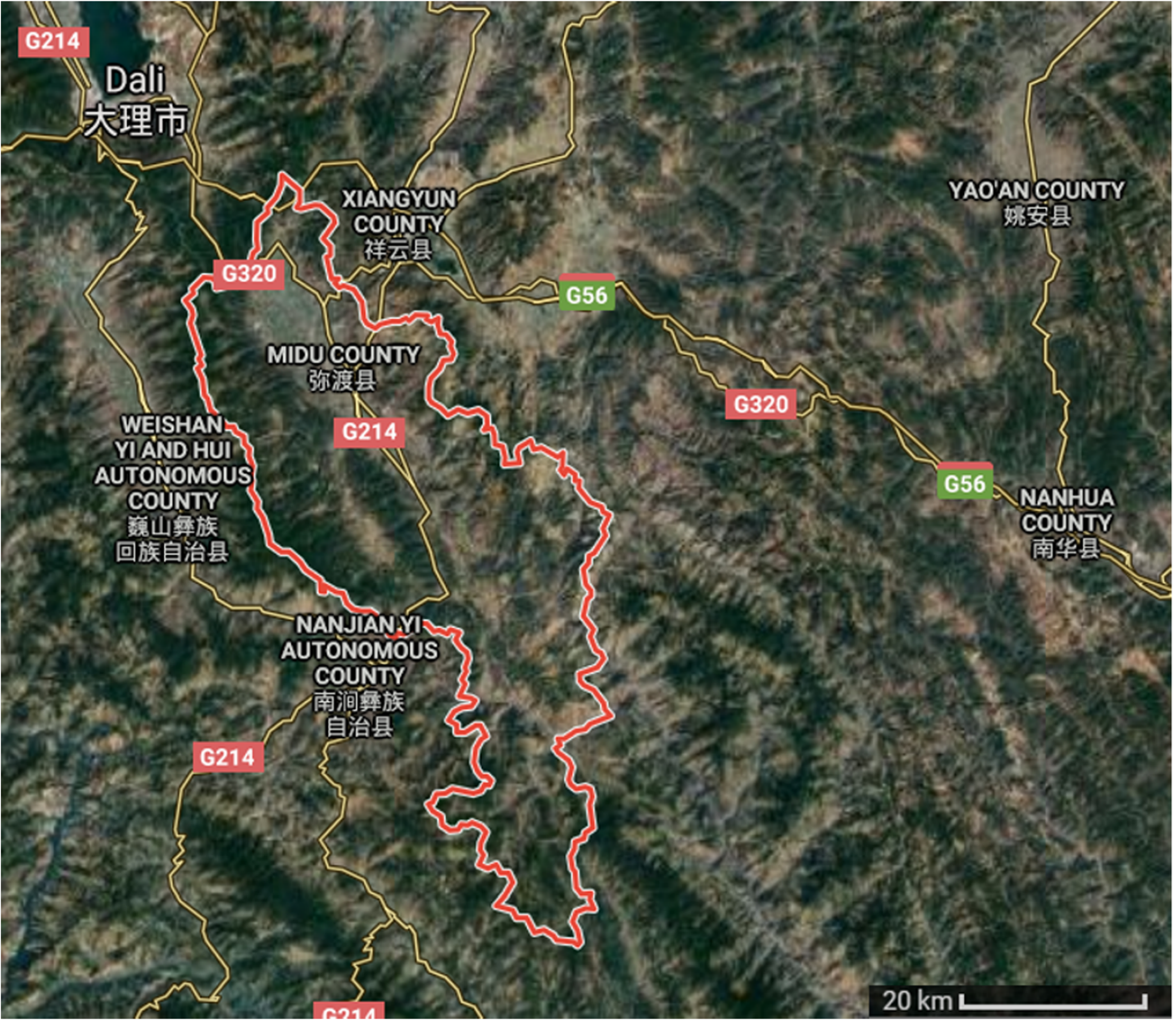
Map of the area from the Dali Autonomous Prefecture in Yunnan Province where mushrooms were collected; 25° 20′ 34″ N 100° 29′ 35″ E (Google maps)

Mushrooms were collected from the geochemically anomalous region from the Midu County (Dali Bai Autonomous Prefecture) located in the west-central region of Yunnan province in China in 2018 (Fig. 1). Species such as *Baorangia bicolor* (Kuntze) G. Wu, Halling & Zhu L. Yang (earlier called *Boletus bicolor* Peck) and the sample whole (fresh) weight was 433 g (*n* = 21) for uncooked and 402 g (*n* = 22) for stir-fried fruiting bodies; *Boletus bainiugan* Dentinger (earlier called in SW China *Boletus edulis* Bull*.*) weighing 280 g (*n* = 5) and 292 g (*n* = 6); *Butyriboletus roseoflavus* (earlier called *Boletus speciosus* Forst.) weighing 299 g (*n* = 5) and 342 g (*n* = 5); *Retiboletus griseus* (Frost) Manfr. Binder & Bresinsky (earlier called *Boletus griseus* Frost) weighing 366 g (*n* = 8) and 341 g (*n* = 8); *Rugiboletus extremiorientalis* (Lj.N. Vassiljeva) G. Wu & Zhu L. Yang (earlier called *Leccinum extremiorientale* (Lj.N. Vassiljeva)) weighing 440 g (*n* = 12) and 312 g (*n* = 12), and *Sutorius magnificus* (W.F. Chiu) G. Wu & Zhu L. Yang (earlier called *Boletus magnificus* W.F. Chiu) weighing 154 g (*n* = 6) and 312 g (*n* = 7) (Cui et al. 2016; Feng et al. 2012) were sampled.

### Cooking

Fruiting bodies that were randomly selected for stir-frying pools were collected at the same time and location as the raw counterpart pools. Each collected fruiting body within a pool was individually cleaned from foreign debris, and the morphological parts (cap and stem) were separated. All specimens not subjected for stir-frying were sliced and dried for 24 h to a constant mass at 65 °C in a food dehydrator (Ultra FD1000, Ezidri, Australia), and ground to a fine powder using a clean porcelain mortar and pestle and stored dry in sealed polyethylene bags until analysis.

All specimens subjected for stir-frying were sliced, separately caps and stems, pooled accordingly and stir-fried using one of the traditional Yunnan methods in moderate amount of hot vegetable oil (15 to 55 g and depending on the sample size) in a wok pan for 10 min. After cooking, the excess oil was drained away and the fried mushrooms were cooled and transferred into fresh polyethylene jars (screw capped, 0.5 L), weighted, deep frozen (− 20 °C), freeze-dried for 72 h, then reweighed (to calculate moisture content), homogenized using a blender with steel-less blades and plastic bowl, and kept tightly closed in screw capped polycarbonate jars which were packed individually into sealed polyethylene bags and stored in a refrigerator. Immediately prior to the instrumental analysis, all the fungal materials were deep frozen (− 20 °C) and freeze-dried for 72 h (Labconco Freeze Dry System, Kansas City, MO, USA), so that the activity levels of nuclides were determined in fully dehydrated materials.

### Analysis

Activity concentrations of ^137^Cs and ^40^K were determined using a gamma spectrometer with a coaxial HPGe detector and with a relative efficiency of 18% and a resolution of 1.9 keV at 1.332 MeV (with associated electronics). Quantitation was carried out using the equation:

1 $$ {A}_{\mathrm{i}}=\frac{N_{\mathrm{i}}}{t\ \varepsilon \left(\mathrm{E}\right)\ y\ } $$

where *N*_i_ is the number of counts after background correction, ε(E) is the detector efficiency for photons with energy E, *y* is the emission probability, and *t* is the measurement time in seconds.

All measurements were preceded by a background measurement (time 80,000 s or 250,000 s), and background counts were subtracted (using the GENIE 2000 program). The lower limit of detection was at 0.10 Bq kg^−1^ dry biomass (db). The equipment was calibrated using a multi-isotope standard, and the method was fully validated (Falandysz et al. 2019d; Wang et al. 2015). The reference material “Standard solution of gamma emitting isotopes, code BW/Z-62/27/07” produced at the IBJ-Świerk near Otwock in Poland was used for preparing reference samples for equipment calibration. The radionuclides used in the multicomponent reference solution during equipment calibration were ^137^Cs at 1.5% and several other nuclides with an approximation uncertainty level at 0.80-2.1%.

The same geometry of cylindrical dishes with 40 mm diameter (as used for the measurement of collected samples) was used for reference samples during equipment calibration. Calibration was carried out using standards with a density of approximately 1 g cm^3^(liquid) with different heights: 3, 6, 9, 15, and 25 mm, which allows the selection of the appropriate calibration for samples of different thickness layer. All numerical data obtained were recalculated for dehydrated fungal material (freeze-dried), and all data were decay-corrected back to the time of sampling. Concentrations of total (stable) K (Table 2) were calculated from the ^40^K activity concentration (mean value) in natural K, which is in the range 27.33 to 31.31 Bq g^−1^ of K (Samat et al. 1997).

## Results and discussion

### ^137^Cs, ^40^K, and K in dried raw mushrooms

The activity concentrations of ^137^Cs in pooled samples of caps of the species were in the range 14 ± 1 Bq kg^−1^ db in *Retiboletus griseus* to 34 ± 2 Bq kg^−1^ db in *Rugiboletus extremiorientalis*. Values of ^137^Cs in the pooled samples of stems were from 16 ± 1 Bq kg^−1^ db in *Baorangia bicolor* and *Boletus bainiugan* to 23 ± 1 Bq kg^−1^ db in *Rugiboletus extremiorientalis*. The mean activity concentration in the whole fruiting bodies in all six species was 18 ± 4 Bq kg^−1^ db (Table 1).

**Table 1:** Values of ^137^Cs and ^40^K activity concentrations (Bq kg^−1^ dry biomass) and concentration of total K (mg kg^−1^ dry biomass) in raw (fresh) and stir-fried (whole weight) mushrooms (mean ± SD)

Species and morphological part of fruitbody	Raw mushrooms			Stir-fried mushrooms		
	^137^Cs	^40^K	K	^137^Cs	^40^K	K
*Baorangia bicolor*						
Caps	16 ± 1	890 ± 96	32,000 ± 3400	8.5 ± 0.9	190 ± 51	6800 ± 1800
Stems	16 ± 1	550 ± 83	19,000 ± 2900	18 ± 2	200 ± 100	7300 ± 3800
Whole	16 ± 1	740 ± 91	26,000 ± 3200	11 ± 2	190 ± 80	7000 ± 2800
*Boletus bainiugan*						
Caps	19 ± 2	940 ± 120	34,000 ± 4200	18 ± 2	480 ± 110	17,000 ± 3900
Stems	16 ± 1	730 ± 89	26,000 ± 3300	12 ± 1	250 ± 78	9100 ± 2800
Whole	17 ± 1	870 ± 100	29,000 ± 3700	15 ± 2	350 ± 95	12,000 ± 3300
*Butyriboletus roseoflavus*						
Caps	18 ± 2	1000 ± 100	37,000 ± 3500	18 ± 2	300 ± 120	11,000 ± 4300
Stems	17 ± 2	630 ± 100	23,000 ± 2500	17 ± 2	200 ± 110	7000 ± 4000
Whole	18 ± 2	830 ± 100	30,000 ± 3100	18 ± 2	250 ± 120	9000 ± 4200
*Retiboletus griseus*						
Caps	14 ± 1	1100 ± 93	40,000 ± 3300	10 ± 1	460 ± 59	17,000 ± 2100
Stems	17 ± 2	1000 ± 110	37,000 ± 3800	23 ± 2	530 ± 150	20,000 ± 5200
Whole	15 ± 1	1100 ± 100	39,000 ± 3500	14 ± 2	480 ± 110	18,000 ± 3600
*Rugiboletus extremiorientalis*						
Caps	34 ± 2	960 ± 97	34,000 ± 3500	18 ± 1	350 ± 100	12,000 ± 3800
Stems	23 ± 1	630 ± 30	23,000 ± 2500	14 ± 1	250 ± 58	9100 ± 2100
Whole	27 ± 2	750 ± 54	27,000 ± 2800	15 ± 1	280 ± 70	10,000 ± 2700
*Sutorius magnificus*						
Caps	18 ± 2	960 ± 140	34,000 ± 4900	23 ± 2	480 ± 140	17,000 ± 5100
Stems	19 ± 2	500 ± 110	19,000 ± 3000	20 ± 2	200 ± 130	7100 ± 4800
Whole	18 ± 2	760 ± 120	27,000 ± 3900	22 ± 2	350 ± 130	12,000 ± 4900
Overall mean (caps)	20 ± 7	970 ± 71	35,000 ± 3000	16 ± 5	380 ± 120	13,000 ± 4200
Overall mean (stems)	18 ± 3	670 ± 180	24,000 ± 7000	17 ± 4	270 ± 130	10,000 ± 5000
Overall mean (whole)	18 ± 4	840 ± 120	30,000 ± 4400	16 ± 4	320 ± 130	11,000 ± 4600

Notes: Data on percentage of moisture content of the fresh and cooked mushrooms, fresh and dry biomass share between caps and stems of the fruiting bodies, percentage of hidden oil sorbed by fried mushrooms, percentage of moisture loss by cooked mushrooms due to dehydration, and proportion between fresh mushrooms biomass and oil sorbed in the course of frying on are given in detail in an earlier report (Falandysz et al. 2019c)

The data obtained for ^137^Cs confirm that wild mushrooms foraged from the Yunnan province in China show low contamination levels, and the activity concentrations were within the range of values reported so far both in wild and cultivated mushrooms from southwestern Asia (Falandysz et al. 2015, 2016, 2017. 2018; Tuo et al. 2014; Wang et al. 2015). However, ^137^Cs at a somewhat greater activity concentration, i.e., 210 Bq kg^−1^ db (range 150 ± 9 to 340 ± 22 Bq kg^−1^ db) has been found in the shaggy (scaly or woolly) chanterelle *Gomphus floccosus* (Schv.) Sing in one study (Tuo et al. 2014), current name *Turbinellus floccosus* (Schwein.) Earle ex Giachini and Castellano (Index Fungorum 2020). Also, mushrooms from the subalpine region of the Minya Konka (Mount Gongga or Gongga Shan) in the Eastern Tibetan Plateau, showed, on average, higher levels of ^137^Cs than the present study, i.e., in the range 62 ± 6 to 280 ± 150 Bq kg^−1^ db in caps and 62 ± 6 to 400 ± 72 Bq kg^−1^ db in stems (Falandysz et al. 2018).

^40^K in caps was in the range 890 ± 96 Bq kg^−1^ db (*Baorangia bicolor*) to 1100 ± 93 Bq kg^−1^ db (*Retiboletus griseus*) and in stems 500 ± 110 Bq kg^−1^ db (*Sutorius magnificus*) to 1000 ± 110 Bq kg^−1^ db (*Retiboletus griseus*). The whole fruiting bodies showed activity concentrations of ^40^K in the range from 740 Bq kg^−1^ db (*Baorangia bicolor*) to 1100 Bq kg^−1^ db (*Retiboletus griseus*), and the mean value for all six species was 840 ± 120 Bq kg^−1^ db. The distribution of ^137^Cs and ^40^K between morphological parts of the fruiting bodies (Quotient_Cap/Stem_) was similar (Q_C/S_ ~ 1) in *Boletus bainiugan*, *Rugiboletus extremiorientalis*, and *Retiboletus griseus*, but favored the caps (mean Q_C/S_ 1.6) in *Baorangia bicolor*, *Butyriboletus roseoflavus*, and *Sutorius magnificus*. The overall mean distribution ratios for all the fruiting bodies sampled were Q_C/S_ 1.1 ± 0.2 for ^137^Cs and Q_C/S_ 1.5 ± 0.3 for ^40^K.

The total K content determined was in the range of 32,000 ± 3400 mg kg^−1^ db (*Baorangia bicolor*) to 40,000 ± 3300 mg kg^−1^ db (*Retiboletus griseus*) in the caps, and 19,000 ± 3000 mg kg^−1^ db (*Baorangia bicolor* and *Sutorius magnificus*) to 37,000 ± 3800 mg kg^−1^ db (*Retiboletus griseus*) in the stems. Soil mushrooms, including species of the family *Boletaceae*, are rich in K. In the present study, the total K contents in the whole fruiting bodies were in the range 26,000 ± 3200 mg kg^−1^ db (*Baorangia bicolor*) to 39,000 ± 3500 (*Retiboletus griseus*) mg kg^−1^ db, and the overall mean was 30,000 ± 4400 mg kg^−1^ db.

Data obtained on K in species in the present study agree with the results obtained for *B. edulis* that grows in European forests, which contained the element in the range 25,000 ± 4000 to 29,000 ± 3000 mg kg^−1^ db (caps) and 16,000 ± 4000 to 20,000 ± 3000 mg kg^−1^ db (stems) (Falandysz et al. 2011; Frankowska et al. 2010; Zhang et al. 2010). The small fluctuation in the concentration range of K in species such as *Boletus edulis* from a range of the geographical regions is relatively narrow. It is thought to be a result of the essentiality and homeostatic regulation of this major metallic element absorbed from soil by mushrooms (Stijve 1996).

### ^137^Cs, ^40^K, and K in stir-fried mushrooms

The activity concentrations of ^137^Cs were roughly the same in both dehydrated samples, stir-fried and raw mushrooms, while the contents of ^40^K and stable K were around 2- to 3-fold smaller in stir-fried than raw produce (Table 1). In detail, the overall mean of the activity concentration of ^137^Cs in the stir-fried mushrooms (whole fruiting bodies) for six species was 16 ± 4 Bq kg^−1^ db (range 11 ± 2 to 22 ± 2 Bq kg^−1^ db), and of ^40^K, was 320 ± 92 Bq kg^−1^ db (range 190 ± 80 to 480 ± 110 Bq kg^−1^ db). The overall mean content of K in stir-fried mushrooms was 11,000 ± 4600 mg kg^−1^ db, and the range for all species was 7000 ± 2800 to 18,000 ± 3600 mg kg^−1^ db (Table 1). Clearly, the content of ^40^K and K in the stir-fried mushrooms when normalized to dry biomass basis was far less than in dehydrated raw mushrooms (humidity in the range from 88.2% in *Rugiboletus extremiorientalis* to 91.7% in *Retiboletus griseus*) (Falandysz et al. 2019c). The vegetable oil used in stir-frying of mushrooms showed activity concentration of ^137^Cs in 0.24 ± 0.05 Bq per g, and was free of K.

Data obtained (Table 1) shows higher retention of ^137^Cs than of ^40^K (and stable K) in the course of stir-frying. This may be due to the difference in the distribution of Cs and K in cell structures and their binding sites. Potassium is a major monovalent element that is found in high concentrations in mushrooms and is a key component of the cellular protoplasm. Breakout from cell walls due to the effects of high-temperature cell shrinkage during the course of stir-drying can favor the release of K, but can have a lower effect on Cs. Also a preferable leaching of K from cells into the residual oily phase is possible. Stir-frying causes partial dehydration of cooked foodstuffs and resulting in an increase in the proportion of dry matter in a mushroom meal but also of some less volatile or non-volatile compounds of the metallic elements, e.g., mercury (Falandysz et al. 2019a, c). The volume or proportion of oil per unit of stir-fried mushrooms depends on the cooking practice or on local tradition in the different regions of Yunnan. In the present study, fried mushrooms lost from 36 to 55% of the original humidity and absorbed almost all the entire volume of the oil added (from 24 to 32 g of oil per 100 g) to raw (fresh) mushrooms, leaving a small residual volume (0.82 to 5.7 g per dish) (Falandysz et al. 2019c).

Data from the literature shows that when frying (flat pan) mushrooms *Cantharellus cibarius* and *Boletus edulis*, a substantial portion of ^137^Cs leaked out into the oily residue, which could be discarded, but the possible intake of ^137^Cs with fried mushroom meals has not been estimated (Steinhauser and Steinhauser 2016). Wetting or wet-cleaning of *Cantharellus cibarius* and *Boletus edulis* from foreign debris before frying could increase the loss of ^137^Cs into the oily residue. The rate of leaching of ^137^Cs in washed chanterelles was 19 to 29 %, and from 2 to 16 % from mushrooms that were cleaned dry. The corresponding rates for *Boletus edulis* were 6 % and 0 % respectively (Steinhauser and Steinhauser 2016).

In an earlier study, Kenigsberg et al. (1996) reported that frying of mushrooms decreased the activity concentration of ^137^Cs by 70 % (probably when calculated on a dry biomass basis in relation to the activity concentration in dehydrated uncooked mushrooms) but without information on the mushroom species studied or on the intake of the nuclide based on the whole weight of the fried mushroom meal.

### ^137^Cs, ^40^K, and K in mushroom meals, possible intake, and exposure to radioactivity

All of the species surveyed in this study were edible, both stems and caps of the fruiting bodies. Thus, data on ^137^Cs, ^40^K, and K in raw mushrooms and mushroom meals were recalculated for appropriate meal portions for stems and caps, and are expressed for the whole fruiting bodies on a whole weight basis (Table 2).

**Table 2:** Values of ^137^Cs and ^40^K mean activity concentrations (Bq kg^−1^ whole weight) and K content (mg kg^−1^ whole weight) in the composite samples of the whole fruiting bodies of a raw and stir-fried mushrooms

Species (whole fruiting bodies)	Raw mushrooms			Stir-fried mushrooms		
	^137^Cs	^40^K	K	^137^Cs	^40^K	K
*Baorangia bicolor*	1.8	82	2900	6.1	100	3900
*Boletus bainiugan*	1.6	82	2700	6.0	140	4800
*Butyriboletus roseoflavus*	1.9	88	3200	7.7	110	3800
*Retiboletus griseus*	1.2	91	3200	6.0	200	7700
*Rugiboletus extremiorientalis*	3.2	88	3200	7.0	130	4700
*Sutorius magnificus*	1.9	90	3200	9.4	150	5100
Overall mean	1.9 ± 0.6	87 ± 4	3100 ± 200	7.0 ± 1.2	140 ± 32	5000 ± 1300

The raw mushrooms and stir-fried mushroom meals on a whole weight showed activity concentrations of ^137^Cs respectively in the range from 1.2 to 3.2 Bq kg^−1^ ww (mean 1.9 ± 0.6 Bq kg^−1^ ww) and from 6.0 to 9.4 Bq kg^−1^ ww (mean 7.0 ± 1.2 Bq kg^−1^ ww). Evidently, when expressed on a whole (produce) weight basis, the stir-fried meals showed on average around 3.5-fold greater activity concentration of ^137^Cs when compared with raw mushrooms.

In the case of ^40^K, the activity concentration in raw mushrooms was in the range of 82 to 91 Bq kg^−1^ ww (mean 87 ± 4 Bq kg^−1^ ww), and in the stir-fried mushroom meals, in the range of 100 to 200 Bq kg^−1^ ww (mean 140 ± 32 Bq kg^−1^ ww). Analogically, the content of stable K in fresh mushrooms was in the range from 2700 to 3200 mg kg^−1^ ww (mean 3100 ± 200 mg kg^−1^ ww) and in cooked produce, from 3800 to 7700 mg kg^−1^ ww (mean 5000 ± 1300 mg kg^−1^ ww).

Potassium (both ^40^K and total K) were slightly enriched in the whole weight stir-fried mushroom meals when compared with the whole weight raw mushrooms (Table 2), and this outcome was very different when compared with data expressed on dry biomass basis (Table 1). Thus, it can be expected that in the course of stir-frying, a large portion of potassium escaped out of the mushroom meals, while for ^137^Cs, the rate of loss was lower.

It has been estimated from a previous report (Falandysz et al. 2019c) that during the mushroom foraging season in southwestern China, certain elements of the population can consume a 100-g portion of stir-fried mushrooms daily, per capita, over a period of several weeks. On this basis, the estimated average intake of ^137^Cs, ^40^K, and total K with a single meal per capita would be 0.70 ± 0.12 Bq, 14 ± 3 Bq, and 500 ± 130 mg, and 4.9 ± 0.9 Bq, 96 ± 23 Bq, and 3500 ± 910 mg respectively for weekly intake. The estimated daily intake of ^137^Cs, ^40^K, and total K normalized to per kilogram of body mass (bm; typical weight of 60 kg per individual) was 12 ± 2 mBq, 230 ± 52 mBq, and 8.3 ± 2.2 mg respectively, and the corresponding weekly intake was 82 ± 14 mBq, 1600 ± 360 mBq, and 58 ± 15 mg respectively (Table 3).

**Table 3:** Estimated intake of ^137^Cs, ^40^K, and K from the stir-fried (whole weight) mushroom meals and exposure doses from ^137^Cs and ^40^K decay for adult person*

Species (whole fruiting bodies)	Daily and weekly intake per capita			Daily and weekly intake per kg^-1^ bm			Daily and weekly effective dose, per capita		Daily and weekly effective dose, per kg bm	
	100 g × 1 meal//100 g × 7 meals			100 g × 1 meal//100 g × 7 meals						
	^137^Cs	^40^K	K	^137^Cs	^40^K	K	^137^Cs	^40^K	^137^Cs	^40^K
	Bq		mg	mBq		mg	μSv		μSv	
*Baorangia bicolor*	0.61//4.3	10//70	390//2700	10//72	170//1200	6.5//45	0.0079//0.056	0.062//0.43	0.00013//0.00093	0.0010//0.0072
*Boletus bainiugan*	0.60//4.2	14//98	480//3400	10//70	230//1600	8.0//56	0.0078//0.055	0.087//0.61	0.00013//0.00091	0.0014//0.010
*Butyriboletus roseoflavus*	0.77//5.4	11//77	380//2700	13//90	180//1300	6.3//44	0.010//0.070	0.068//0.48	0.00017//0.0012	0.0011//0.0080
*Retiboletus griseus*	0.70//4.9	13//91	470//3300	12//82	220//1500	7.8//55	0.0091//0.064	0.081//0.56	0.00015//0.0011	0.0013//0.0094
*Rugiboletus extremiorientalis*	0.60//4.2	20//140	770//5400	10//70	330//2300	13//90	0.0078//0.055	0.12//0.87	0.00013//0.00091	0.0021//0.014
*Sutorius magnificus*	0.94 // 6.6	15//100	510//3600	16//110	250//1700	8.5//59	0.012//0.086	0.093//0.65	0.00020//0.0014	0.0016//0.011
Mean	0.70 ± 0.12//4.9 ± 0.9	14 ± 3//96 ± 23	500 ± 130//3500 ± 910	12 ± 2//82 ± 14	230 ± 52//1600 ± 360	8.3 ± 2.2//58 ± 15	0.0091 ± 0.015//0.064 ± 0.011	0.085 ± 0.019//0.60 ± 0.14	0.00015 ± 0.00003//0.0011 ± 0.0002	0.0014 ± 0.0004//0.010 ± 0.002

Notes: ^137^Cs and ^40^K (Bq kg^-1^ whole (wet) weight); K (mg kg^-1^ whole (wet) weight)

*Asian adult (60 kg body mass)

The average effective daily and weekly doses of gamma radioactivity received per capita from ^137^Cs and ^40^K contained in stir-fried mushroom meals in this study have been estimated as 0.0091 ± 0.015 μSv and 0.064 ± 0.011 μSv for ^137^Cs and 0.085 ± 0.019 μSv and 0.60 ± 0.14 μSv for ^40^K (Table 3). The average daily and weekly effective doses received by consumers from ^137^Cs and ^40^K expressed per kilogram of body mass have been assessed as 0.00015 ± 0.00003 μSv and 0.0011 ± 0.0007 μSv for ^137^Cs and 0.0014 ± 0.0004 μSv and 0.010 ± 0.002 μSv for ^40^K (Table 3). Therefore, on a single meal or annual basis, a mean radioactivity dose from natural ^40^K (0.51 μSv per kilogram of body mass) in around 9.3-fold exceeded the dose obtained for artificial ^137^Cs (0.055 μSv), which both are very low.

Adequate potassium daily intake for adults has been set for 4700 mg (NIH 2019), and a serving composed of 100 g stir-fried (whole weight) mushroom meals containing between 380 and 770 mg of K (mean 500 ± 130 mg; Table 2) and assuming that absorption rate by body is 85 to 90% classifies the mushroom meals in this study among a good potential dietary source of potassium. Mushrooms that are conserved are much poorer source of potassium (Pankavec et al. 2019) than the stir-fried products.

## Conclusions

Examination of the parallel batches of the same species and geographical origin both uncooked and stir-fried mushrooms showed that the activity concentrations of ^137^Cs were roughly the same in both dehydrated samples, stir-fried, and raw mushrooms, while the contents of ^40^K and stable K were around 2- to 3-fold smaller in stir-fried than raw produce. The stir-fried mushroom meals on a whole (wet) weight exhibited on average around 3.5-fold greater activity concentration of ^137^Cs when compared with raw mushrooms. The ^137^Cs enrichment in cooking, and less of ^40^K (in a whole (wet) weight basis for the meal), confronted with the results for dried raw and fried mushrooms show the direct correlation with loss of mass (largely moisture during the cooking procedure) but not much of ^137^Cs and ^40^K; therefore, activity concentrations of both nuclides in stir-fried mushrooms increase. Hence, exposure to radiocaesium from meals made of the stir-fried mushrooms substantially contaminated with this nuclide can be a more significant source than speculated earlier. However, edible wild mushrooms from Yunnan are little contaminated with radiocaesium. An assessed, the mean radioactivity dose from natural ^40^K in around 9.3-fold exceeded the dose obtained for artificial ^137^Cs from stir-fried mushroom meals in Yunnan, which both were very low doses. The effect of the stir-frying process on ^137^Cs and other mineral constituent content in mushroom meals has to be clarified from the ongoing studies.
